# Maternal Nonstandard Work Schedules and Breastfeeding Behaviors

**DOI:** 10.1007/s10995-016-2233-4

**Published:** 2017-01-13

**Authors:** Afshin Zilanawala

**Affiliations:** Department of Epidemiology and Public Health, University College London, 1-19 Torrington Place, London, WC1E 6BT UK

**Keywords:** Breastfeeding, Nonstandard work, Cohort studies, Maternal employment

## Abstract

*Objectives *Although maternal employment rates have increased in the last decade in the UK, there is very little research investigating the linkages between maternal nonstandard work schedules (i.e., work schedules outside of the Monday through Friday, 9–5 schedule) and breastfeeding initiation and duration, especially given the wide literature citing the health advantages of breastfeeding for mothers and children.* Methods* This paper uses a population-based, UK cohort study, the Millennium Cohort Study (n = 17,397), to investigate the association between types of maternal nonstandard work (evening, night, away from home overnight, and weekends) and breastfeeding behaviors.* Results* In unadjusted models, exposure to evening shifts was associated with greater odds of breastfeeding initiation (OR 1.71, CI 1.50–1.94) and greater odds of short (OR 1.55, CI 1.32–1.81), intermediate (OR 2.01, CI 1.64–2.47), prolonged partial duration (OR 2.20, CI 1.78–2.72), and prolonged exclusive duration (OR 1.53, CI 1.29–1.82), compared with mothers who were unemployed and those who work other types of nonstandard shifts. Socioeconomic advantage of mothers working evening schedules largely explained the higher odds of breastfeeding initiation and duration.* Conclusions* Socioeconomic characteristics explain more breastfeeding behaviors among mothers working evening shifts. Policy interventions to increase breastfeeding initiation and duration should consider the timing of maternal work schedules.

## Significance

Nonstandard work schedules may have adverse consequences for maternal and child health. Although, literature has established that employment in the first year of life is related to breastfeeding initiation and duration, research has not examined the influence of nonstandard work on breastfeeding behaviors. The current study finds evening shifts to be associated with greater odds of breastfeeding initiation and longer breastfeeding durations. Higher odds are accounted for by advantaged socioeconomic conditions. Intervention strategies promoting breastfeeding may consider maternal nonstandard work schedules.

## Introduction

Over the past half century, a large proportion of women with children have entered the labor force in the United Kingdom (UK). This is commensurate with a growth in the service sector. These two economic changes are credited to the growing phenomenon of nonstandard work (NW) (e.g., working evenings, nights, or weekend shifts). In the UK, recent data finds that nearly 30% of all workers work shifts that are usually in the evening, night, or are rotating shifts and about 1/5 of all workers work on the weekends (Presser et al. 2008). About a quarter of employed mothers work evenings, nights, or rotating shifts and about 18% of these mothers work on the weekends.

Despite the prevalence of working outside standard hours, no research has examined the association between mother’s NW schedules and breastfeeding imitation and duration. Compelling evidence highlights the protective effect of breastfeeding for children and mothers, such as attainment of gross motor milestones (Sacker et al. 2006), fewer infections (Hetzner et al. 2009), improved cognitive development (Quigley et al. 2012), lower rates of child obesity (Grummer-Strawn and Mei 2004), lower risk of postpartum depression (Borra et al. 2014), and lower rates of breast and ovarian cancer for the mother (Labbok 2001). Given the high prevalence of NW schedules in the UK and the transition to a “24/7” service economy (Presser 2003) and the potential advantages of breastfeeding, it is important to examine whether NW influences breastfeeding behaviors.

Prior evidence has established that employment in the first year of life is related to breastfeeding initiation and duration (Fein and Roe 1998; Hawkins et al. 2007; Mandal et al. 2010). This research has found returning to work early and working full-time are linked to lower odds of breastfeeding initiation and shorter breastfeeding durations (Mandal et al. 2010). Mothers who are in part-time employment or are unemployed do not differ in their probability of breastfeeding initiation (Hawkins et al. 2007). Financial constraints and supportive work arrangements partially explain these differences.

The link between NW and children’s health could be mediated by parental depression, less effective parenting, and poor family functioning (Strazdins et al. 2006). Women working NW schedules experience more sleep deprivation and work/family conflicts (Maume and Sebastian 2012). Stress, depression, and physiological consequences of such schedules may in turn adversely affect children’s health and development (Heymann and Earle 2001). Infants have basic caregiving needs and demand time and effort from a primary caregiver to form a secure attachment. A mother’s ability to initiate and sustain breastfeeding during this critical period of a child’s life may be hampered by her poor biological functions, stress, and fatigue resulting from working nonstandard hours (Li et al. 2008).

Studies about the factors associated with NW and with breastfeeding behaviors can shed light on potential correlates and selection factors of any observed link between NW schedules and breastfeeding patterns. Mothers who had a low-birth weight infant, are an ethnic minority, are younger (<20 years old), and did not have a college education have lower rates of breastfeeding initiation (Ryan et al. 2006). Equally women with higher education or who are White are less likely to work NW schedules (McMenamin 2007). With regard to *types* of NW schedules, mothers who work evening or night shifts have lower educational attainment, work fewer hours per week, and earn less than those who work standard hours (Wight et al. 2008). Indeed, the probability a mother works night hours (compared to day or evening shifts) increases alongside increases in the number of children in the household.

The probability of continuing to breastfeed after returning to work may depend on a mother’s occupation or socioeconomic status. More than one-third of workers in service occupations work nonstandard schedules, including half of workers in the food preparation and protective services industries, whereas less than 10% of workers in management and professional jobs do so (McMenamin 2007). Mothers in professional occupations may have more control in their workplace environment and flexibility in their schedules to integrate the demands of work and the needs of their breastfeeding infants (Visness and Kennedy 1997). Therefore, the potential implications of NW are greater for breastfeeding continuously.

The reasons why new mothers work NW schedules may also influence her breastfeeding behaviors. Choosing a NW schedule may be preferable for a mother if she can rely on “split-shift” parenting with another caregiver (Presser 2003). Evidence suggests parents may use a tag-team parenting strategy that integrates their work schedules so that at least one parent or caregiver is available (Hattery 2001; Täht and Mills 2012). Thus, NW schedules may be a preferable choice if it allows a mother to be available during the day to attend to breastfeeding schedules. Other mothers may choose such schedules if higher wages are offered. However, U.S. Census data suggests that constraints or requirements of the job dictated working NW shifts rather than preferences (McMenamin 2007).

Factors that are otherwise typically unobserved in secondary data may also influence continued successful breastfeeding. There may be concerns about sufficient support from employers and co-workers, and real or perceived milk supply (Arthur et al. 2003; Shealy et al. 2005). A further obstacle is a workplace program that supports and encourages breastfeeding; without one, a new mother returning to work may abandon her infant feeding efforts (Ryan et al. 2006). Postpartum events, such as physical recovery from childbirth, changes in role identities, and infant health problems, may also prove to be barriers to continuous breastfeeding (Chatterji and Frick 2005).

Family income and structure may moderate the influence of maternal NW on breastfeeding behaviors. NW schedules are more common among low-income and less educated individuals (Presser 2003). Mothers who have a lower economic wellbeing may be less able to respond to the challenges of NW schedules and have limited resources to support and sustain breastfeeding. This may include, for example, purchasing services that promote breastfeeding, such as breast pumps or lactation consultants. Similarly, mothers who have a married or cohabiting partner may have more time and energy to breastfeed resulting from the greater social and emotional support and shared household responsibilities from a partner (Guzzo and Lee 2008; Kiernan and Pickett 2006).

Previous literature has collapsed different types of NW schedules (i.e., evening, night, weekend) into one category of NW making it difficult to establish which schedules influence children’s health and development and family processes (Li et al. 2014). The type of NW schedule may affect family processes differently (Han and Waldfogel 2007). Although evidence suggests mothers who work at night spend more time with their children as compared to mothers who work at other hours of the day (Wight et al. 2008), such schedules may induce insufficient sleep (Kantermann et al. 2010) or fatigue (Heymann and Earle 2001) and reduce quality parenting and effective breastfeeding behaviors. Although, studies have highlighted the importance of disaggregating NW schedules to elucidate which schedules are influential to children’s health (Dunifon et al. 2013; Li et al. 2014), no research has examined whether types of maternal NW schedules are associated with breastfeeding behaviors.

This study takes into consideration previous literature demonstrating the difficulty combining employment and breastfeeding (Hawkins et al. 2007) and the potential work-family obstacles upon returning to paid employment postpartum (Rippeyoung and Noonan 2012). Given the differential effects on family and child wellbeing, the current investigation considers the types of NW. Regarding the hypothesized direction of any observed associations between NW and breastfeeding behaviors, it is difficult to anticipate the direction by types of NW (i.e., evening, night, and other shifts) because each work situation has dual-potential to constrain or facilitate breastfeeding behaviors. For instance, evening or night work schedules make it difficult for a mother to schedule consistent breastfeeding patterns. However, at the same time, these shifts may allow a mother to breastfeed during the day and/or pump milk, which can be supplied by another caregiver. NW schedules may help a mother respond to infant feeding needs more flexibly. Where there is an observed association between NW schedules and breastfeeding initiation and duration, it is anticipated that these linkages would be mediated by socio-economic and demographic factors. Previous literature has emphasized the greater likelihood of working NW schedules among lower income mothers’ due to job requirements rather than personal preferences. Additionally, lower income mothers may have more limited resources to mitigate the challenges of balancing work and family demands.

The current study will extend the literature by using a nationally representative cohort study to examine the linkages between maternal nonstandard work schedules and the likelihood of breastfeeding initiation and duration. The following research questions will be investigated:: (a) Are particular types of maternal NW schedules (evenings, nights, and weekends) associated with breastfeeding initiation and duration?; and (b) What maternal demographic, socioeconomic, and psychosocial factors help explain any observed differences? It is important to note that this analysis is descriptive and does not assess causal associations between NW schedules and breastfeeding behaviors. Although prior research uses cross-sectional analyses to interrogate the link between maternal employment and breastfeeding behaviors (Hawkins et al. 2007; Ryan et al. 2006), the current study is the first to investigate the relationship between types of NW schedules and breastfeeding patterns. Understanding whether there are variations in breastfeeding behaviors among mothers who work such schedules is important as public health policy makers consider ways to encourage breastfeeding.

## Methods

### Data

This study uses a national cohort study that follows children from birth to middle childhood: the Millennium Cohort Study (MCS). The MCS is a cohort study of 18,552 children born in the United Kingdom between 2000 and 2002. It is representative of infants who were alive and residing in the United Kingdom at 9 months of age and who were eligible for Child Benefit (a nearly universal monetary benefit) (Plewis et al. 2004). The sample was clustered at the electoral ward level, with an oversample of disadvantaged residential areas and areas with a high proportion of ethnic minority residents. The first interviews were at 9 months of age, and follow up sweeps were conducted at ages 3, 5, 7, and 11. The analyses in this study use data from the 9-month interview. During this interview, questions were asked about infant feeding practices, child and maternal characteristics, socioeconomic circumstances, and psychosocial factors. The analytic sample is 17,397 and excludes multiple births (n = 256) and observations with incomplete information on covariates (n = 842). As a secondary analysis of anonymized data, this study is conducted in accord with prevailing ethical principles.

#### Breastfeeding

A bivariate indicator for breastfeeding initiation was created from the question, “Did you ever try to breastfeed?”. Breastfeeding duration was constructed from questions about the age of the infant when last given breast milk and when formula, other types of milk, and solid foods were first given to the child. From these questions, partial and exclusive breastfeeding behaviors were derived and categorized in 2 month bands: never breastfed; short duration (terminated breastfeeding before 2 months); intermediate duration (terminated breastfeeding after 2 months but before 4 months of age); prolonged partial (breastfed for 4 months or more supplementing with solids or formula beginning before 4 months); and prolonged exclusive (breastfed for 4 months or more supplementing with solids or formula beginning at 4 months or later) (Sacker et al. 2006). These categories were informed by the United Kingdom infant feeding guidelines at the time of the survey, which recommended exclusive breastfeeding for 4–6 months.

#### Nonstandard Work

Information on maternal NW was collected at 9 months. Employed parents are asked to report the frequency they worked each type of NW schedule: evening (between 6 pm and 10 pm), night (10 pm–7 am), away from home overnight, and weekends. Mothers were allowed to choose more than one schedule, making the NW schedule categories nonmutually exclusive. The response categories are “every week”, “at least once a month”, “less than once a month”, and “never”. For each type of NW schedule, responses were collapsed into a binary variable in which respondents were coded as 1 if they worked a particular schedule every week or 0 if they responded otherwise (i.e., at least once a month or less frequently). Types of NW were four nonmutually exclusive groups: mothers working evenings, nights, overnights, and weekends. Respondents were categorized as working a *standard* schedule if they were employed but indicated they did not have any of the NW schedules described. In regression analyses, the reference category is not working at the time of the survey (i.e., not working a standard schedule or any of the nonstandard schedules). The measures of NW capture exposure to types of NW, which may be in combination with other types of NW schedules or experienced in isolation. The categorization employed in this analysis follows recent empirical evidence linking the exposure of maternal NW schedules and child wellbeing (Dunifon et al. 2013).

#### Covariates

Child and maternal characteristics are: child age, gender, mother’s age at birth, mother’s ethnicity (White, Mixed, Indian, Pakistani, Bangladeshi, Black Caribbean, Black African, or Other) (Kelly et al. 2006), and smoking status (Never smoked, Used to smoke regularly, 1–10 per day, or ≥11 per day). Socioeconomic characteristics include maternal education (NVQ equivalence scale: NVQ5 Degree or higher, NVQ4 Diploma, NVQ3 A/AS levels, NVQ2 >4 GCSEs, NVQ1 <5 GCSEs, Overseas qualification, or None)^1^) (McIntosh and Steedman 1999), two parent household indicator, and equivalized household income in quintiles. Psychosocial factors assessed childcare arrangements (Nursery, Parental care, Relatives or neighbors, Nanny or childminder, or On leave/Other), mother’s Malaise Inventory Score, and mother’s postnatal attachment score. The Malaise Inventory is a shortened version of the original 24-item scale measuring psychological distress; the self-completion questionnaire has been used in population studies (Rodgers et al. 1999; Rutter et al. 1970). Maternal attachment to her child is assessed using 6 Likert items, adopted from the Condon Maternal Attachment Questionnaire (Condon and Corkindale 1998).

### Statistical Analysis

A series of regression models examined the independent contribution of child, maternal, socioeconomic, and psychosocial factors on breastfeeding. All models adjust for child age and gender. Model 1 presents with unadjusted estimates of NW. Model 2 adjusts for child and maternal characteristics; Model 3 adjusts for socioeconomic factors; Model 4 adjusts for psychosocial factors; Model 5 simultaneously controls for all covariates. In sensitivity analyses not shown, covariates were added simultaneously instead of independently; for example, socioeconomic factors were adjusted for alongside child and maternal characteristics. Additionally, reverse ordering of covariate adjustments were conducted. These extra analyses revealed no differences to findings reported below.

For models predicting breastfeeding initiation, multivariate logistic regressions were run and odds ratios are reported. For models predicting breastfeeding duration, multinomial logistic regression models were run and odds ratios are reported. In regression analyses, the reference category is not working at the time of the survey (i.e., not working a standard schedule or any of the nonstandard schedules). Post-hoc tests were conducted to ascertain whether coefficients on types of NW schedules were statistically different from each other and from standard shifts. All analyses adjust for oversampling and the stratified sample design.

## Results

Table 1 presents the descriptive statistics of the study variables, including the breastfeeding measures, nonstandard employment characteristics, and covariates for the sample.

**Table 1:** Descriptive statistics

Variable	Mean or %	Standard deviation
Ever breastfed	69.6	
Breastfeeding duration		
Never breastfed	30.4	
Short duration	29.3	
Intermediate duration	7.4	
Prolonged partial	14.4	
Prolonged exclusive	18.6	
Work schedules		
Standard only	28.1	
Mother worked evenings	16.4	
Mother worked nights	5.0	
Mother worked weekends	11.1	
Mother worked overnights	1.2	
Mother unemployed	51.3	
Child and maternal characteristics		
Child is male	51.5	
Mother’s age at birth	28.8	5.8
Mother’s ethnicity		
White	89.9	
Mixed	0.9	
Indian	1.8	
Pakistani	2.6	
Bangladeshi	0.8	
Black Caribbean	1.1	
Black African	1.4	
Other	1.5	
Smoking status		
Never smoked	51.4	
Smoked regularly but no longer	19.8	
Up to 10 per day	18.0	
More than 11 per day	10.8	
Socioeconomic characteristics		
Mother’s education		
None	11.8	
Overseas	2.2	
NVQ1	8.3	
NVQ2	30.1	
NVQ3	14.4	
NVQ4	29.5	
NVQ5	3.7	
Two parent household	85.8	
Equivalized household income		
Lowest quintile	19.3	
Second quintile	19.7	
Third quintile	20.0	
Fourth quintile	20.5	
Highest quintile	20.5	
Psychosocial characteristics		
Child care arrangements		
Nursery	6.2	
Parental care	16.9	
Relatives or neighbors	17.4	
Nanny or childminder	5.4	
On leave/other	54.1	
Malaise score [0–9]	1.6	1.7
Postnatal attachment scale [8–29]	24.0	2.9
*n*	17,263	

All means are weighted by Millennium cohort study sample weights. Sample size is unweighted and exclusive to singleton births and respondents who are natural mothers. Higher scores on malaise reflect more psychological distress and higher scores on attachment are more positive

Nearly 70% of mothers in the sample breastfed their child. Thirty percent of mothers ceased breastfeeding before 2 months and one-third of mothers breastfed for at least 4 months. About half of mothers were not working at the time of the survey, and nearly 30% were working a standard shift (28.1%). Twenty percent of the sample worked a nonstandard shift; 16.4% reported working evenings, 5% worked nights, 11.1% worked weekends, and 1.2% worked overnights (NW categories were not mutually exclusive). Among those who were employed, 58% worked a standard shift and 42% worked a nonstandard shift; 34% worked evenings, 10% worked nights, 23% worked weekends, and 2% worked overnights.

Mothers were on average 29 years old at the time of the child’s birth. Half of mothers had never smoked but nearly 30% were current smokers (28.8%). One-third of sample mothers had NVQ4 or higher (equivalent to college degree or higher) and the majority were married at the time of the child’s birth. Of the nearly 45% of children who were in child care, the most common arrangements were parental and relatives or neighbor care.

Table 2 illustrates the odds ratios (OR) and 95% confidence intervals (CIs) from logistic regressions predicting breastfeeding initiation by maternal NW shifts.

**Table 2:** Odds ratios (95% CI) of breastfeeding initiation by maternal nonstandard work schedules (N = 17,263)

Work schedules	Model 1	Model 2: Model 1 + child and maternal characteristics	Model 3: Model 1 + socioeconomic	Model 4: Model 1 + psychosocial	Model 5: fully adjusted
Mother worked evenings	1.71*** (1.50–1.94)	1.52*** (1.33–1.73)	0.93 (0.81–1.06)	1.66*** (1.40–1.97)	1.02 (0.85–1.22)
Mother worked nights	0.92^a,b^ (0.72–1.17)	0.98 (0.75–1.27)	1.11 (0.86–1.43)	0.94 (0.73–1.20)	1.05 (0.81–1.38)
Mother worked weekends	0.99^a,b^ (0.85–1.15)	1.03 (0.88–1.20)	0.84 (0.71–1.01)	1.04 (0.88–1.22)	0.92 (0.77–1.11)
Mother worked overnights	1.26 (0.85–1.86)	0.98 (0.66–1.46)	0.94 (0.62–1.41)	1.24 (0.83–1.86)	0.84 (0.56–1.26)
Standard only	1.76*** (1.58–1.95)	1.36*** (1.22–1.51)	0.79*** (0.70–0.89)	1.69*** (1.44–1.99)	0.87 (0.73–1.05)

All analyses are weighted using sample weights from the Millennium cohort study

*p < 0.05, **p < 0.01, ***p < 0.001

^a^Nonstandard shift significantly different from evening shift at *p* < 0.001

^b^Nonstandard shift significantly different from standard shift at *p* < 0.001

In Model 1, mothers working evenings had 70% greater odds of breastfeeding initiation, compared with mothers who were unemployed (OR 1.71, CI 1.50–1.94). The greater likelihood of breastfeeding initiation from exposure to working evening shifts was significantly different from that of working night or weekend shifts. Exposure to other types of maternal NW schedules was not associated with breastfeeding initiation. Adjustment for child and maternal characteristics attenuated the odds for mothers working evening shifts (Model 2). Adjustment for socioeconomic characteristics attenuated the odds to nonsignificant levels (Model 3). Psychosocial factors did not explain the greater likelihood of breastfeeding initiation (Model 4). In fully adjusted models, the increased odds of breastfeeding associated with exposure to evening shifts were no longer apparent (OR 1.02, CI 0.85–1.22).

In an examination of individual markers of socioeconomic status (analyses not shown), separate adjustments were made for maternal education, family structure, and equivalized income. Maternal education and equivalized income attenuated estimates the most and to nonsignificant levels. The association between evening schedules and breastfeeding initiation did not differ by maternal education. There was modest evidence of moderation by income.

Table 3 presents risk ratios for breastfeeding duration, as compared to never breastfed, across NW schedules.

**Table 3:** Relative risk ratios (95% CI) predicting breastfeeding duration by maternal nonstandard work schedules (N = 17,263)

Breastfeeding duration (ref: never breastfed)	Model 1	Model 2: Model 1 + child and maternal characteristics	Model 3: Model 1 + socioeconomic	Model 4: Model 1 + psychosocial	Model 5: fully adjusted
Short duration					
Mother worked evenings	1.55*** (1.32–1.81)	1.50*** (1.27–1.76)	1.04 (0.88–1.22)	1.33** (1.10–1.62)	1.00 (0.81–1.22)
Mother worked nights	0.92^a,e^ (0.70–1.22)	0.95 (0.71–1.26)	1.02 (0.77–1.35)	0.94 (0.71–1.24)	1.00 (0.75–1.33)
Mother worked weekends	1.06^a,e^ (0.89–1.27)	1.07 (0.90–1.29)	0.92 (0.76–1.12)	0.99 (0.81–1.20)	0.90 (0.73–1.11)
Mother worked overnights	1.14 (0.69–1.87)	1.03 (0.64–1.68)	0.96 (0.59–1.55)	1.13 (0.69–1.85)	0.92 (0.57–1.47)
Standard only	1.59*** (1.41–1.78)	1.43*** (1.27–1.60)	0.92 (0.81–1.04)	1.30** (1.09–1.56)	0.87 (0.71–1.06)
Intermediate duration					
Mother worked evenings	2.01*** (1.64–2.47)	1.81*** (1.47–2.22)	1.07 (0.87–1.33)	1.89*** (1.38–2.58)	1.15 (0.83–1.58)
Mother worked nights	1.05^a,e^ (0.70–1.59)	1.12 (0.73–1.72)	1.27 (0.84–1.94)	1.08 (0.72–1.62)	1.21 (0.79–1.86)
Mother worked weekends	1.14^a,e^ (0.87–1.49)	1.19 (0.91–1.57)	0.95 (0.71–1.26)	1.16 (0.85–1.58)	1.02 (0.75–1.40)
Mother worked overnights	1.07^b,f^ (0.53–2.16)	0.84 (0.41–1.72)	0.83 (0.40–1.71)	1.05 (0.52–2.15)	0.72 (0.35–1.50)
Standard only	2.08*** (1.74–2.49)	1.64*** (1.36–1.98)	0.89 (0.72–1.08)	1.90*** (1.35–2.66)	0.95 (0.67–1.36)
Prolonged partial duration					
Mother worked evenings	2.20*** (1.78–2.72)	1.82*** (1.47–2.26)	0.96 (0.76–1.20)	2.27*** (1.70–3.02)	1.16 (0.87–1.54)
Mother worked nights	0.86^a,e^ (0.60–1.22)	0.95 (0.65–1.39)	1.11 (0.76–1.61)	0.89 (0.62–1.27)	1.07 (0.72–1.57)
Mother worked weekends	0.92^a,e^ (0.72–1.17)	0.97 (0.76–1.25)	0.75* (0.58–0.97)	1.02 (0.79–1.31)	0.90 (0.69–1.18)
Mother worked overnights	1.17^a,f^ (0.67–2.04)	0.81 (0.45–1.44)	0.80 (0.45–1.40)	1.16 (0.66–2.04)	0.66 (0.36–1.19)
Standard only	2.06*** (1.78–2.38)	1.42*** (1.22–1.65)	0.73*** (0.62–0.86)	2.10*** (1.63–2.72)	0.89 (0.68–1.16)
Prolonged exclusive duration					
Mother worked evenings	1.53*** (1.29–1.82)	1.24* (1.03–1.48)	0.65*** (0.54–0.78)	1.79*** (1.43–2.25)	0.86 (0.68–1.09)
Mother worked nights	0.91^a,c,e^ (0.67–1.24)	1.02 (0.73–1.42)	1.23 (0.89–1.71)	0.92 (0.67–1.26)	1.14 (0.80–1.62)
Mother worked weekends	0.86^a,d,e^ (0.70–1.06)	0.92 (0.74–1.15)	0.72** (0.57–0.90)	1.04 (0.82–1.31)	0.92 (0.71–1.18)
Mother worked overnights	1.65* (1.03–2.65)	1.12 (0.67–1.88)	1.10 (0.65–1.86)	1.65* (1.02–2.67)	0.91 (0.53–1.55)
Standard only	1.71*** (1.49–1.95)	1.13 (0.98–1.31)	0.58*** (0.50–0.68)	2.10*** (1.69–2.60)	0.83 (0.65–1.05)

All analyses are weighted using sample weights from the Millennium cohort study

*p < 0.05, **p < 0.01, ***p < 0.001

^a^Nonstandard shift significantly different from evening shift at *p* < 0.05

^b^Nonstandard shift significantly different from evening shift at *p* < 0.10

^c^Nonstandard shift significantly different from overnight shift at *p* < 0.10

^d^Nonstandard shift significantly different from overnight shift at *p* < 0.05

^e^Nonstandard shift significantly different from standard shift at *p* < 0.05

^f^Nonstandard shift significantly different from standard shift at *p* < 0.10

In Model 1, mothers’ evening work, compared to unemployment, was associated with greater odds of short breastfeeding duration (OR 1.55, CI 1.32–1.81), intermediate duration (OR 2.01, CI 1.64–2.47), prolonged partial duration (OR 2.20, CI 1.78–2.72), and prolonged exclusive duration (OR 1.53, CI 1.29–1.82). The coefficients on evening work differed significantly from those of night shifts, weekend shifts, and overnight schedules. However, evening work did not differ from standard schedule work in predicting breastfeeding duration. Exposure to overnight schedules, compared to nonwork, was associated with 65% greater odds of prolonged exclusive duration. The coefficient on overnight work differed significantly from those of night and weekend shifts. In contrast, exposure to other types of maternal nonstandard schedules (i.e., nights and weekends) was not associated with breastfeeding duration. Adjustment for child and maternal characteristics reduced the odds for mothers working evening schedules and attenuated estimates for overnight schedules to non-significance (Model 2). Socioeconomic characteristics reduced the odds for overnight and evening schedules to non-significant levels with the exception of odds of prolonged exclusive duration for mothers working evening shifts (OR 0.65, CI 0.54–0.78; Model 3). In analyses not shown, both income and maternal education reduced estimates the most and to non-significance. Adjustment for psychosocial factors made little difference to estimates (Model 4). Fully adjusted models showed no significant differences in the odds of breastfeeding duration by maternal NW schedules (Model 5).

A series of sensitivity tests (results not shown) were run controlling for health endowment at birth, as proxied by low birth-weight and gestational age at birth, in unadjusted models. Previous studies have underscored differences in breastfeeding behaviors by birth-weight and preterm and term children (Quigley et al. 2012; Ryan et al. 2006). The initial estimates for breastfeeding initiation and duration were not altered. An additional analysis was conducted to consider the length of maternity leave. Mothers who intend to initiate breastfeeding or to breastfeed for longer may arrange for longer maternity leave (Chatterji and Frick 2005). Adjustment for maternal leave time did not significantly alter NW schedule estimates in Models 1, for both initiation and duration. Significance and substantive findings were not influenced by length of leave time.

## Discussion

In a period in which maternal employment rates have been increasing in the UK (Office for National Statistics 2011), it is salient to understand the implications of employment on maternal and child health. In particular, examining breastfeeding as an outcome is paramount given its potential social and economic benefits (Ball and Bennett 2001; Rippeyoung and Noonan 2012), and its association with better child cognitive and behavioral development (Quigley et al. 2012). The timing of work schedules is a key factor in influencing maternal and child wellbeing and family functioning (Li et al. 2014). Working nonstandard hours may complicate work and family life, and may constrain time with children that relates to their health and development.

This descriptive study examined the link between exposure to types of maternal NW schedules and breastfeeding initiation and duration. At 9 months, the 20% of the sample worked a nonstandard shift and evening shifts were the most common type of nonstandard schedule. Nearly 40% of employed mothers worked a nonstandard shift. Evidence suggests maternal evening shifts were associated with 70% greater odds of breastfeeding initiation compared with mothers who were unemployed (unadjusted estimates). Mothers working in the evening were more likely to breastfeed than mothers who worked night or weekend shifts. There was incremental increasing likelihood of breastfeeding duration corresponding to longer durations of breastfeeding for evening schedules: exposure to evening work, compared to unemployment, was associated with greater odds of short, intermediate, and prolonged breastfeeding duration (unadjusted estimates). The higher odds of breastfeeding duration were different from those of night, weekend, and overnight work schedules. These significant associations between evening schedules and breastfeeding initiation and duration were explained by socioeconomic selection factors as operationalized here. Of such markers, equivalized household income and maternal education did most to explain the higher odds of breastfeeding initiation and duration; socioeconomic advantage of mothers working evening schedules was linked to greater likelihood of breastfeeding initiation and duration.

With respect to both breastfeeding initiation and duration, the unadjusted and fully adjusted models showed that no other type of NW schedule (that is, nights, weekends, or overnight shifts) was consistently associated with breastfeeding behaviors. It may be that mothers who have evening shifts have positive breastfeeding experiences and continue to breastfeed for longer duration while maintaining attachment to the labor force at shifts that are compatible with their breastfeeding practices. Alongside these positive experiences, supportive work arrangements may influence a mother’s decision to breastfeed and to continue for longer durations (Hawkins et al. 2007).

Results from this study support evidence finding lower income households, less educated, and non-professional mothers are less likely to breastfeed compared to higher income, more educated, and professional working women (Chatterji and Frick 2005; Haider et al. 2003; Ryan et al. 2006). The literature on work characteristics and their influence on initiation and duration of breastfeeding generally finds working is negatively related to breastfeeding; full-time employment is linked to lower breastfeeding rates as compared to mothers’ unemployment (Hawkins et al. 2007; Ryan et al. 2006) and returning to work within 3 months of delivery is associated with lower likelihood of breastfeeding (Chatterji and Frick 2005). Only one study examined the exposure to nonstandard employment on breastfeeding initiation and reported a positive association between an aggregate measure of nonstandard employment and breastfeeding initiation (Hawkins et al. 2007), but the authors did not examine breastfeeding duration. In the current study, disaggregating the types of nonstandard schedules revealed important differences between shifts. Suggestive descriptive evidence shows that exposure to evening schedules, as compared to unemployment, is related to greater likelihood of breastfeeding initiation and longer duration of breastfeeding. These associations stand in stark contrast to findings on higher initiation rates and breastfeeding intensity for unemployed mothers. The influence of maternal NW on maternal and child health may differ by the timing of such schedules (Dunifon et al. 2013; Wight et al. 2008). Evening schedules, as compared to irregular or night shifts, may be associated with less sleep deficit and in turn may afford mothers fewer time constraints and greater availability to care for and breastfeed their children. Therefore, evening schedules may play a unique role in maternal and child health. This highlights the need for future studies to disaggregate nonstandard schedules to capture both advantages and disadvantages conferred to different schedules.

With these data it is difficult to ascertain why long-duration breastfeeders work evening schedules after childbirth compared to mothers working other nonstandard schedules or mothers not in the labor force. The directionality between working evening schedules and breastfeeding behaviors is indiscernible. Mothers may choose to work evening schedules before deciding to breastfeed and to breastfeed for a particular duration; alternatively, mothers may decide to breastfeed before deciding work schedules. In the MCS, nearly 70% of mothers returning to employment postpartum did so for financial reasons (Hawkins et al. 2007). Women who are less educated have fewer employment opportunities and are overrepresented in jobs with nonstandard hours. Evidence from American census data suggests that more than half of individuals working nonstandard shifts do so in response to family and job constraints rather than personal preferences (McMenamin 2007). Descriptive evidence in the current study suggests that mothers who are combining long duration breastfeeding with nonstandard employment is counter to the ideology of intensive mothering (Hays 1998). This framework suggests family-oriented women privilege their children’s health and needs over their own and thus may sacrifice their working lives. Long-duration breastfeeding could be a proxy for intensive mothering (Rippeyoung and Noonan 2012); however, findings from this paper suggest mothers are *coupling* breastfeeding with NW schedules. This could be due to positive breastfeeding experiences, such as supportive employer arrangements, a private place to pump milk, a place to store expressed milk, or other strategies (Gatrell 2007; Hawkins et al. 2007). It is important for future research to investigate workplace arrangements and structural supports for mothers who are combining their NW schedules and breastfeeding practices.

The current study examined exposure to any nonstandard schedule on breastfeeding practices at 9 months. It will be important for future research to interrogate NW at different durations and intensity. Data was limited to retrospective interviews with mothers at 9 months postpartum but evidence finds that maternal recall of breastfeeding initiation and duration is valid and reliable (Li et al. 2005). Although it is conceivable that mothers may have given socially desirable answers or poorly recalled their breastfeeding experiences, and such imprecisions could have created measurement error. For example, there may be misclassification of breastfeeding in the shortest breastfeeding categories. The shortest breastfeeding category is heterogeneous and includes infant feeding for a few days or a couple of weeks, thus resulting in an underestimate or overestimate of the linkages between NW schedules and breastfeeding. Further, the data do not include information on types of formula milk. These are important areas to expand upon in future data collection endeavors in birth cohort studies.

An important strength of the current study was that analyses adjusted for a number of potential socioeconomic, maternal, and psychosocial factors that may have accounted for the linkages between NW and breastfeeding practices. Although MCS data have rich information on socioeconomic factors, it is still possible there are important omitted variables, such as wealth, that may elucidate the relationships between NW schedules and breastfeeding patterns. A further empirical problem arises from the lack of information on intention to breastfeed. Although a mother’s intention may change before or after the birth of a child, a reasonable surrogate of intention is maternity leave; indeed initial results presented here were not confounded by length of leave. Lastly, the findings from this study do not accurately portray the complex set of decision making and tradeoffs mothers make when integrating work schedules and breastfeeding. These dynamics may be further understood using qualitative data that collects information on how mothers manage NW schedule constraints and breastfeeding behaviors.

In summary, this is the first UK study to add to the growing literature on the prevalence of nonstandard schedules among mothers of a contemporary cohort of children. The descriptive finding that mother’s evening schedules are positively associated with breastfeeding initiation and longer breastfeeding durations has important policy implications. UK employment policies have encouraged maternal employment (Dolton and Smith 2011) while Department of Health initiatives are facilitating breastfeeding initiation, and particularly for disadvantaged groups, who are disproportionally low-income and less educated (Executive 2003). Future public health strategies can incorporate NW schedules in policies supporting women who aim to combine working and breastfeeding practices.

## Footnotes

1 The equivalent educational categories are College degree or higher (NVQ4 and 5), Some college (NVQ3), High school (NVQ2), and Less than high school (NVQ1) (Zilanawala et al. 2015).

## References

[CR1] Arthur C, Saenz R, Replogle W (2003). The employment-related breastfeeding decisions of physician mothers. Journal of the Mississippi State Medical Association.

[CR2] Ball TM, Bennett DM (2001). The economic impact of breastfeeding. Pediatric Clinics of North America.

[CR3] Borra C, Iacovou M, Sevilla A (2014). New evidence on breastfeeding and postpartum depression: The importance of understanding women’s intentions. Maternal and Child Health Journal.

[CR4] Chatterji P, Frick KD (2005). Does returning to work after childbirth affect breastfeeding practices?. Review of Economics of the Household.

[CR5] Condon JT, Corkindale CJ (1998). The assessment of parent-to-infant attachment: Development of a self-report questionnaire instrument. Journal of Reproductive and Infant Psychology.

[CR6] Dolton, P., & Smith, J. A. (2011). *The impact of the UK new deal for lone parents on benefit receipt*, Discussion Paper.

[CR7] Dunifon R, Kalil A, Crosby DA, Su JH (2013). Mothers’ night work and children’s behavior problems. Developmental Psychology.

[CR9] Fein SB, Roe B (1998). The effect of work status on initiation and duration of breast-feeding. American Journal of Public Health.

[CR10] Gatrell CJ (2007). Secrets and lies: breastfeeding and professional paid work. Social Science & Medicine.

[CR11] Grummer-Strawn LM, Mei Z (2004). Does breastfeeding protect against pediatric overweight? Analysis of longitudinal data from the centers for disease control and prevention pediatric nutrition surveillance system. Pediatrics.

[CR12] Guzzo KB, Lee H (2008). Couple relationship status and patterns in early parenting practices. Journal of Marriage and Family.

[CR13] Haider SJ, Jacknowitz A, Schoeni RF (2003). Welfare work requirements and child well-being: Evidence from the effects on breast-feeding. Demography.

[CR14] Han W-J, Waldfogel J (2007). Parental work schedules, family process, and early adolescents’ risky behavior. Children and Youth Services Review.

[CR15] Hattery A (2001). Tag-team parenting: Costs and benefits of utilizing nonoverlapping shift work in families with young children. Families in Society.

[CR16] Hawkins SS, Griffiths LJ, Dezateux C, Law C (2007). Maternal employment and breast-feeding initiation: Findings from the Millennium Cohort Study. Paediatric and Perinatal Epidemiology.

[CR17] Hays S (1998). The cultural contradictions of motherhood.

[CR18] Hetzner NMP, Razza RA, Malone LM, Brooks-Gunn J (2009). Associations among feeding behaviors during infancy and child illness at two years. Maternal and Child Health Journal.

[CR19] Heymann SJ, Earle A (2001). The impact of parental working conditions on school-age children: The case of evening work. Community, Work & Family.

[CR20] Kantermann T, Juda M, Vetter C, Roenneberg T (2010). Shift-work research: Where do we stand, where should we go?. Sleep and Biological Rhythms.

[CR01] Kelly YJ, Watt RG, Nazroo JY (2006). Racial/ethnic differences in breastfeeding initiation and continuation in the United Kingdom and comparison with findings in the United States. Pediatrics.

[CR21] Kiernan K, Pickett KE (2006). Marital status disparities in maternal smoking during pregnancy, breastfeeding and maternal depression. Social Science & Medicine.

[CR22] Labbok MH (2001). Effects of breastfeeding on the mother. Pediatric Clinics of North America.

[CR23] Labour Force Survey, Office for National Statistics. (2011). Mothers in the Labour Market, 2011.

[CR24] Li J, Johnson SE, Han W-J, Andrews S, Kendall G, Strazdins L, Dockery A (2014). Parents’ nonstandard work schedules and child well-being: A critical review of the literature. The Journal of Primary Prevention.

[CR25] Li J, Kendall G, Henderson S, Downie J, Landsborough L, Oddy W (2008). Maternal psychosocial well-being in pregnancy and breastfeeding duration. Acta Paediatrica.

[CR26] Li R, Scanlon KS, Serdula MK (2005). The validity and reliability of maternal recall of breastfeeding practice. Nutrition Reviews.

[CR27] Mandal B, Roe BE, Fein SB (2010). The differential effects of full-time and part-time work status on breastfeeding. Health Policy.

[CR28] Maume DJ, Sebastian RA (2012). Gender, nonstandard work schedules, and marital quality. Journal of Family and Economic Issues.

[CR29] McIntosh S, Steedman H (1999). Qualifications in the United Kingdom 1985–1999.

[CR30] McMenamin TM (2007). Time to work: Recent trends in shift work and flexible schedules, A. Monthly Labor Review.

[CR8] National Health Service Executive. (2003). *Improvement expansion and reform: The next 3 years: Priorities and planning framework 2003–06*. NHS.

[CR31] Plewis I, Calderwood L, Hawkes D, Hughes G, Joshi H (2004). *Millennium cohort study: Technical report on sampling*..

[CR32] Presser HB (2003). *Working in a 24*/*7 economy: Challenges for American families*.

[CR33] Presser HB, Gornick JC, Parashar S (2008). Gender and nonstandard work hours in 12 European countries. Monthly Labor Review.

[CR34] Quigley MA, Hockley C, Carson C, Kelly Y, Renfrew MJ, Sacker A (2012). Breastfeeding is associated with improved child cognitive development: A population-based cohort study. The Journal of Pediatrics.

[CR35] Rippeyoung PL, Noonan MC (2012). Is breastfeeding truly cost free? Income consequences of breastfeeding for women. American Sociological Review.

[CR36] Rodgers B, Pickles A, Power C, Collishaw S, Maughan B (1999). Validity of the Malaise Inventory in general population samples. Social Psychiatry and Psychiatric Epidemiology.

[CR37] Rutter M, Tizard J, Whitmore K (1970). Education, health and behaviour.

[CR38] Ryan AS, Zhou W, Arensberg MB (2006). The effect of employment status on breastfeeding in the United States. Women’s Health Issues.

[CR39] Sacker A, Quigley MA, Kelly YJ (2006). Breastfeeding and developmental delay: Findings from the millennium cohort study. Pediatrics.

[CR40] Shealy KR, Li R, Benton-Davis S, Grummer-Strawn LM (2005). The CDC guide to breastfeeding interventions.

[CR41] Strazdins L, Clements MS, Korda RJ, Broom DH, D’Souza RM (2006). Unsociable work? Nonstandard work schedules, family relationships, and children’s well-being. Journal of Marriage and Family.

[CR42] Täht K, Mills M (2012). Nonstandard work schedules, couple desynchronization, and parent–child interaction a mixed-methods analysis. Journal of Family Issues.

[CR43] Visness CM, Kennedy KI (1997). Maternal employment and breast-feeding: findings from the 1988 National Maternal and Infant Health Survey. American Journal of Public Health.

[CR44] Wight VR, Raley SB, Bianchi SM (2008). Time for children, one’s spouse and oneself among parents who work nonstandard hours. Social Forces.

[CR45] Zilanawala A, Davis-Kean P, Nazroo J, Sacker A, Simonton S, Kelly Y (2015). Race/ethnic disparities in early childhood BMI, obesity and overweight in the United Kingdom and United States. International Journal of Obesity.

